# Newly Diagnosed IDH-Wildtype Glioblastoma and Temporal Muscle Thickness: A Multicenter Analysis

**DOI:** 10.3390/cancers13225610

**Published:** 2021-11-10

**Authors:** Tim Wende, Johannes Kasper, Gordian Prasse, Änne Glass, Thomas Kriesen, Thomas M. Freiman, Jürgen Meixensberger, Christian Henker

**Affiliations:** 1 Department of Neurosurgery, University Hospital Leipzig, 04103 Leipzig, Germany; johannes.kasper@medizin.uni-leipzig.de (J.K.); juergen.meixensberger@medizin.uni-leipzig.de (J.M.); 2 Department of Neuroradiology, University Hospital Leipzig, 04103 Leipzig, Germany; gordian.prasse@medizin.uni-leipzig.de; 3 Institute of Biostatistics and Informatics in Medicine, University Medicine Rostock, 18057 Rostock, Germany; aenne.glass@uni-rostock.de; 4 Department of Neurosurgery, University Medical Center Rostock, 18057 Rostock, Germany; thomas.kriesen@med.uni-rostock.de (T.K.); Thomas.freiman@med.uni-rostock.de (T.M.F.); christian.henker@med.uni-rostock.de (C.H.)

**Keywords:** glioblastoma, temporal muscle thickness, survival, prognostic marker

## Abstract

**Simple Summary:**

Cancer associated cachexia and loss of skeletal muscle mass is a negative prognostic marker for survival. Temporal muscle thickness (TMT) is an easily accessible parameter that has been suggested as a prognostic marker in glioblastoma. In this multicenter study we retrospectively analyzed a cohort of 335 patients with newly diagnosed glioblastoma for their overall survival (OS) and TMT. Although previous studies found TMT to be an independent prognostic marker for OS, we could not reproduce these results. Instead, TMT seems to be a surrogate parameter for other epidemiological data.

**Abstract:**

Background: Reduced temporal muscle thickness (TMT) has been discussed as a prognostic marker in IDH-wildtype glioblastoma. This retrospective multicenter study was designed to investigate whether TMT is an independent prognostic marker in newly diagnosed glioblastoma. Methods: TMT was retrospectively measured in 335 patients with newly diagnosed glioblastoma between 1 January 2014 and 31 December 2019 at the University Hospitals of Leipzig and Rostock. The cohort was dichotomized by TMT and tested for association with overall survival (OS) after 12 months by multivariate proportional hazard calculation. Results: TMT of 7.0 mm or more was associated with increased OS (46.3 ± 3.9% versus 36.6 ± 3.9%, *p* > 0.001). However, the sub-groups showed significant epidemiological differences. In multivariate proportional hazard calculation, patient age (HR 1.01; *p* = 0.004), MGMT promoter status (HR 0.76; *p* = 0.002), EOR (HR 0.61), adjuvant irradiation (HR 0.24) and adjuvant chemotherapy (HR 0.40; all *p* < 0.001) were independent prognostic markers for OS. However, KPS (HR 1.00, *p* = 0.31), BMI (HR 0.98, *p* = 0.11) and TMT (HR 1.06; *p* = 0.07) were not significantly associated with OS. Conclusion: TMT has not appeared as a statistically independent prognostic marker in this cohort of patients with newly diagnosed IDH-wildtype glioblastoma.

## 1. Introduction

Cancer associated cachexia leads to the loss of skeletal muscle, and the thickness of skeletal muscle has been established as an independent prognostic marker of overall survival (OS) in metastasizing cancer [1]. In patients with intracerebral tumors, an easily accessible marker for skeletal muscle mass is temporal muscle thickness (TMT) [2]. Recently, TMT has also been discussed as a prognostic marker in newly diagnosed [3,4,5] and progressive glioblastoma (GBM) [6]. Glioblastoma is the most common malignant tumor of the brain, showing an incidence of 5 per 100,000 per year [7]; the median age at diagnosis is 65 years and men are more frequently affected. Despite great efforts in the advancement of treatment, the prognosis remains devastating, and five-year survival is only 3% [8]. First-line treatment of GBM includes micro-surgical resection followed by concomitant chemoradiotherapy [9,10]. However, depending on prognostic factors, patients are stratified in different therapeutic pathways, where chemotherapy or radiotherapy can be administered without the other. For this reason, and to predict overall survival (OS), prognostic biomarkers are necessary. A minor subtype of GBM develops out of a primary less malignant astrocytoma or oligodendroglioma and shows a mutation of the isocitrate dehydrogenase (IDH) gene, and is called secondary or IDH-positive GBM. The majority of GBM are primary and IDH-negative and have a shorter OS. Other established biomarkers are age, Karnofsky Performance Score (KPS), the extent of resection (EOR), and O^6^-methylguanine-DNA methyltransferase (MGMT) promoter methylation status [10,11]. Current studies describe further genetic and imaging biomarkers as well as neurological performance to be relevant for OS prediction [12,13,14]. 

To analyze whether TMT can serve as an independent prognostic marker, as suggested previously, we examined the records of patients with IDH-negative GBM in a retrospective study of two major German University Hospitals.

## 2. Methods

### 2.1. Patient Cohorts

This study was approved by the local ethics committee (336/20-ek). We analyzed medical records of all patients who were newly diagnosed with IDH-wildtype glioblastoma at the University Hospitals of Leipzig or Rostock, Germany, between 1 January 2014 and 31 December 2019. All patients were at least 18 years old and received magnetic resonance imaging (MRI) with contrast enhancement before surgery and within 72 h after surgery. All patients were treated according to the current guidelines for glioma therapy and all cases were discussed in weekly interdisciplinary tumor boards. Patients who did not undergo surgery were excluded. 

We recorded the age at the date of diagnosis, sex, body mass index (BMI), Karnofsky Performance Score (KPS), MGMT status, extent of resection (EOR), and the administration of adjuvant chemoradiotherapy. Here, gross-total resection was defined as EOR over 90%, sub-total resection (STR) as EOR below 90%, excluding biopsy, and biopsy was defined as burr-hole trepanation with needle biopsy. 

Overall survival (OS) was recorded as the time between tumor resection and death. These were assessed on 30 June 2021. If patients lived beyond that date or were lost to follow-up, the date of last contact was implemented as a censored value. 

### 2.2. TMT Measurement

**Figure 1:**
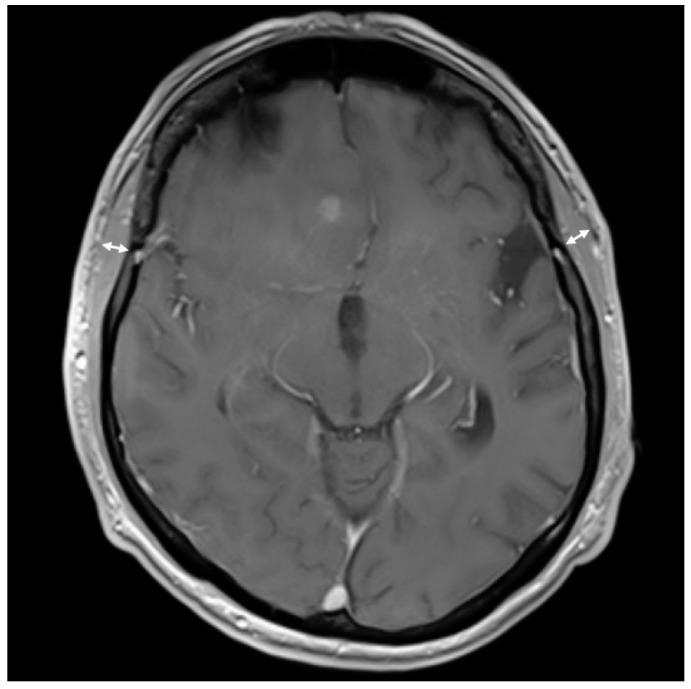
Example of temporal muscle thickness (TMT) measurement in contrast enhanced axial T1 magnetic resonance imaging (MRI) of a 70-year old male patient with an overall survival (OS) of 18 months. Measurement is marked with arrows (right: 7.6 mm, left: 7.8 mm).

For TMT assessment, T1 weighted magnetic resonance images (MRI, 1 mm isovoxel resolution) with gadolinium in axial slides perpendicular to the axis of the temporal muscle were used. An experienced neuroradiologist who was blinded to clinical patient data measured TMT. The measurement was performed on both sides and recorded as mean TMT per patient (Figure 1). 

### 2.3. Statistical Analysis

Statistical analysis was carried out using SPSS statistics software version 24.0.0.2 (IBM, Armonk, NY, USA). Spearman’s rank-order correlation was applied to determine the relationship between BMI and TMT. Receiver operator characteristic (ROC) analysis was performed for TMT. One-year survival was calculated using the Kaplan-Meier estimate, which is given with standard deviation. Statistical significance was determined via log rank testing. Influence on survival probability of continuous and categorized parameters was analyzed via univariate Cox regression. Parameters with p-values below 0.2 in univariate analysis were then implemented into a multivariate proportional hazard calculation. Subgroup comparison of epidemiological data was performed employing Mann-Whitney-U testing. *p*-values below 0.05 were considered to be statistically significant.

## 3. Results

### 3.1. Patient Cohort and Temporal Muscle Thickness

Baseline data is presented in Table 1. During the study period, 335 patients with newly diagnosed glioblastoma were eligible. Concerning epidemiological data such as gender ratio, average age and 12-months survival, the cohort is comparable to larger studies [15]. Average TMT was 7.0 ± 2.1 mm for both sides and average BMI 27.1 ± 5.7 kg/m². Only for values below 30 kg/m² was there a weak correlation between TMT and BMI (Spearman’s ρ 0.247). Otherwise, TMT and BMI did not correlate. Concerning TMT and patient sex, there was also a weak correlation (Spearman’s ρ −0.297).

### 3.2. Temporal Muscle Thickness and Overall Survival

Continuous and categorized parameters were employed in univariate Cox regression to analyze influence on survival probability. Here, increasing KPS (HR 0.98; *p* < 0.001) and TMT (HR 0.92; *p* = 0.004), a positive MGMT methylation status (HR 0.63), gross total resection (HR 0.53), receiving adjuvant irradiation with concomitant temozolomide (HR 0.1) and chemotherapy with temozolomide (HR 0.23; all *p* < 0.001) were significantly correlated with prolonged overall survival while higher patient age was inversely associated with patient survival (HR 1.03; *p* < 0.001). BMI and patient sex did not show statistical significance (Table 2, center column). 

**Figure 2:**
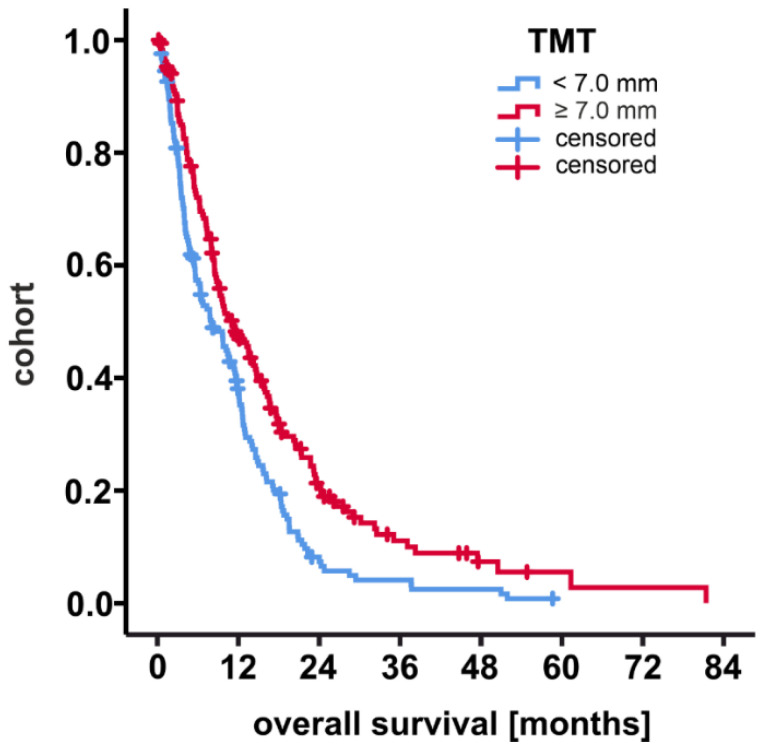
Overall survival and temporal muscle thickness by Kaplan Meier analysis. TMT: temporal muscle thickness.

Since a TMT cutoff could not be calculated by ROC (AUC 0.558), a Kaplan Meier analysis was performed with cohorts stratified by the average TMT from baseline data. Survival curves are shown in Figure 2. Patients with a TMT of 7.0 mm and more had a significantly increased OS (12-months-survival 46.3 ± 3.9%) compared to the corresponding sub-group (12-months-survival 36.6 ± 3.9%, *p* < 0.001 by log-rank test). A comparative sub-cohort analysis, however, revealed that patients with a TMT of 7.0 mm and more were mostly male (as already analyzed before), received GTR as well as adjuvant chemotherapy with temozolomide more often, and had corresponding tumor samples that revealed a methylated MGMT promoter more frequently (Table 3). A subgroup multivariate cox regression analysis of only male or only female patients did not give significant results for TMT, either (*p* = 0.125 and *p* = 0.429, respectively). 

All mentioned parameters were implemented into a multivariate proportional hazard calculation (shown in Table 2, right column). Patient age (HR 1.01; *p* = 0.004), MGMT promoter methylation status (HR 0.76; *p* = 0.002), extent of resection (HR 0.61), adjuvant irradiation (HR 0.24) and adjuvant chemotherapy (HR 0.40; all *p* < 0.001) had independent effects on overall survival. In contrast, BMI (HR 0.98, *p* = 0.11) and TMT (HR 1.06; *p* = 0.07) were not significantly associated with OS.

## 4. Discussion

We present a retrospective analysis of more than 300 patients with newly diagnosed IDH-wildtype glioblastoma from two German university hospitals, investigating the prognostic value of temporal muscle thickness (TMT).

Since a TMT cut-off value could not be defined by ROC analysis, we stratified our patient cohort by the average TMT of 7.0 mm. This cut-off value is comparable with recent studies [4,5,6]. Furthermore, we added the BMI as an additional factor to be considered. In univariate analysis, the difference between the Kaplan Meier estimates of patients with higher and lower TMT values was significantly different. However, there was a bias within other proven prognostic parameters such as EOR, MGMT promoter methylation status and adjuvant therapy regimen. Consequently, multivariate analysis revealed TMT and BMI not to be statistically significant markers for overall survival. Interestingly, there was a weak correlation of TMT and BMI in non-obese patients. This could not be observed in patients with a BMI > 30 kg/m^2^ and might point to a linear relation of overall skeletal muscle mass and body mass, which our data cannot further elaborate. 

It has long been known that cancer, in particular in its metastatic form, leads to cachexia with skeletal muscle atrophy. In these patients, muscular atrophy has proven to be a prognostic marker for survival [1]. In particular, measurement of the TMT has shown to reproduce highly reasonable results and to serve as an easily accessible surrogate of overall skeletal mass, which can be assessed on CT or MR images during routine imaging in cancer staging. The exact underlying mechanism of cancer cachexia, however, is unknown, but metastasizing cancers and chemotherapy toxicities are suspicious of leading to muscle atrophy [16,17]. Taking these results of metastatic cancer studies into consideration, chemotherapy for GBM has a more favorable safety profile than in other cancers, with thrombocytopenia being the main dose-limiting toxicity [9]. Also, metastases of GBM outside the central nervous system are very rare [18], and deterioration is therefore usually caused by decreasing neurological performance rather than systemic effects [19].

Earlier research reported TMT as an independent prognostic marker in GBM after first progression [6], but also found statistical differences in epidemiological data in cohorts stratified by TMT [5]. While this alone sufficiently explains why TMT in our cohort is only a surrogate parameter for epidemiological differences between patients, the statistical significance cannot be reproduced in multivariate analysis. There are several possible explanations for these findings. 

First, we measured TMT before treatment. Therefore, our data rather reflects the overall physical constitution of our patients than the general physical reaction to surgery, chemoradiotherapy or the devastating diagnosis of progressive glioblastoma. This also enabled us to measure TMT bilaterally in all patients, which was not possible in progressive glioblastoma patients, due to temporal muscle atrophy following surgery and irradiation [6]. Also, long-term treatment with corticosteroids that causes skeletal muscle atrophy and compromises survival in glioblastoma, was ruled out in our study design [20,21]. However, if cachexia has been the cause of deterioration directly before death, it would not be detectable in our measurement at the time of diagnosis. Therefore, the time of imaging for skeletal muscle mass may be important.

Second, patients with unknown or mutated IDH-status were excluded from our cohort. Considering IDH-wildtype GBM (now known as Astrocytoma WHO grade IV [10]) as an independent entity, allowed for a more coherent data analysis in comparison to previous works that did not screen for IDH status [3], or included both IDH-mutated and IDH-wildtype GBM [6]. Last, our multi-center cohort is larger than those of single-center studies on newly diagnosed glioblastoma and eliminates single-center effects on overall survival and TMT measurement [3,4,5]. 

The epidemiological data in our cohort is comparable to larger studies [7]. We also found known epidemiological markers for overall survival to be statistically significant in multivariate analysis (Table 2). Specifically, patient age, MGMT promoter methylation status, extent of resection, adjuvant irradiation and adjuvant chemotherapy were independent markers for overall survival [10,11,12,13,14]. These study characteristics emphasize the quality and comparability of our data. The limitations of this study lie within its design, which is of a retrospective nature, and therefore a recruitment bias cannot be fully ruled out. Although TMT was measured as described in other studies, there may be inter-observer variability, which was not ruled out by a further analysis during this study. However, measuring the thickness of the temporal muscle in a defined point along its axis is not prone to large aberrations. 

## 5. Conclusions

In metastasizing cancers, TMT is a well investigated marker for patient survival [22,23]. Although it has been proposed as an independent marker for survival in progressive as well as in newly diagnosed glioblastoma [3,4,5], our multicenter data showed that in patients with newly diagnosed glioblastoma, TMT is only a surrogate parameter for other epidemiological data. Therefore, in newly diagnosed IDH-wildtype glioblastoma, our data cannot support the role of TMT as an independent prognostic marker. 

## Figures and Tables

**Table 1:** Baseline data.

Characteristics		Value
No. of patients		335
sex	male	196
	female	139
average age (years)		66.8 (18–92.7)
average BMI (kg/m²)		27.1 (18–53.8)
average KPS		72.6 (20–100)
average TMT (mm)		7.0 (3–14.9)
MGMT status	positive	108
	negative	137
	unknown	90
extent of resection	biopsy	80
	STR	102
	GTR	153
adjuvant radiotherapy with concomitant temozolomide	w/o	43
	with	292
adjuvant temozolomide	w/o	127
	with	208
12-months survival (%)		42.2 ± 2.2

Average values are given with their range. BMI: body mass index; GTR: gross-total resection; KPS: Karnofsky Performance Scale; MGMT: O^6^-methylguanine DNA methyltransferase; STR: sub-total resection; TMT: temporal muscle thickness.

**Table 2:** Cox Regression.

Variable	Univariate Cox Regression			Multivariate Cox Regression		
	HR	95CI	*p* Value	HR	95CI	*p* Value
Age	1.03	1.02–1.05	*<0.001*	1.01	1.01–1.03	*0.004*
Sex	0.97	0.72–1.17	0.48	-	-	-
Body Mass Index	0.98	0.96–1.01	0.16	0.98	0.96–1.01	0.11
KPS	0.98	0.98–0.99	*<0.001*	1.00	0.99–1.00	0.31
Temporal Muscle Thickness	0.92	0.87–0.97	*0.004*	1.06	1.00–1.14	0.07
MGMT status	0.63	0.54–0.74	*<0.001*	0.76	0.64–0.91	*0.002*
extent of resection	0.53	0.46–0.62	*<0.001*	0.61	0.52–0.72	*<0.001*
adjuvant irradiation with concomitant temozolomide	0.10	0.07–0.15	*<0.001*	0.24	0.16–0.38	*<0.001*
adjuvant temozolomide	0.23	0.18–0.30	*<0.001*	0.40	0.29–0.55	*<0.001*

Italic values indicate statistical significance. HR: Hazard Ratio; MGMT: O^6^-methylguanine DNA methyltransferase; KPS: Karnofsky Performance Scale; 95CI: 95% confidence interval.

**Table 3:** Sub-cohort stratified by TMT.

Characteristics		TMT ≥ 7.0 mm	TMT < 7.0 mm	*p*-Value
No. of patients		171	164	-
sex	male	123	73	*<0.001*
	female	48	91	
average age (years)		66.4 ± 10.2	65.2 ± 11.4	0.749
average BMI (kg/m²)		26.2 ± 5.1	28.0 ± 5.2	0.905
average KPS		70.2 ± 18.9	75.0 ± 17.0	0.308
MGMT status	positive	71	37	*<0.001*
	negative	74	63	
	unknown	26	64	
extent of resection	biopsy	30	50	*0.005*
	STR	51	51	
	GTR	90	63	
adjuvant radiotherapy with concomitant temozolimide	w/o	16	27	0.263
	with	155	137	
adjuvant temozolomide	w/o	49	78	*0.003*
	with	122	86	

*p*-values were calculated via Mann-Whitney U test. Italic values indicate statistical significance. BMI: body mass index; GTR: gross total resection; KPS: Karnofsky Performance Scale; MGMT: O^6^-methylguanine DNA methyltransferase; STR: sub-total resection; TMT: temporal muscle thickness.

## Data Availability

Data of this work is available from the corresponding author upon reasonable request.
